# The Royal Hospital of St. Bartholomew

**Published:** 1909-11-06

**Authors:** 


### Editor's Letter-Box.

### THE ROYAL HOSPITAL OF ST. BARTHOLOMEW.

*To the Editor of* The Hospital.

Sir,—A few months since public attention was directed to the needs of St. Bartholomew's Hospital by a Conference at the Mansion House between the Lord Mayor, the Treasurer of the Hospital, and the Masters of the leading City Guilds, with reference to the financial position of the institution. At that meeting Lord Sandhurst, the Treasurer, explained the exact state of affairs, and the Lord Mayor read the following letter which he had received from Col. Sir Arthur Bigge, namely :—

"Marlborough House, Pall Mall, S.W.,
"April 6, 1909.

"My dear Lord Mayor,—I am directed by the Prince of Wales to inform you that, as President of St. Bartholomew's Hospital, he has lately had an opportunity of learning from Lord Sandhurst, the Treasurer, that there is now an annual deficit of about £12,000 in the working of the hospital, due in a large measure to payment of interest on a capital debt of, approximately, £170,000. His Royal Highness desires me to say that he has received this information with much regret, as he would view with grave concern the necessity for in any degree curtailing the work of what is the only general hospital in the City boundaries. His Royal Highness sincerely trusts that funds may be raised to prevent the possibility of such an unfortunate contingency.

"Yours very truly,
"Arthur Bigge.

"The Right Hon. the Lord Mayor."

For various reasons, but in particular as it was desired not to clash in any way with the annual collection of the Hospital Sunday Fund, it was decided to postpone making a general appeal until the autumn. The time has now arrived when it has become necessary to take steps to meet the grave situation with which St. Bartholomew's is faced, and we desire to urge its unique claims to public sympathy and support.

For nearly 800 years St. Bartholomew's Hospital has carried on, on its present site, a great work : firstly, in the alleviation of the sufferings of the sick, maimed, and diseased poor; and, secondly, in the training of medical practitioners whose services in after life are of incalculable benefit to humanity, rich and poor alike, all over the world.

From time to time the accommodation of the hospital has been increased to meet the demands upon it, and there are now 680 beds. On an average 8,000 patients are admitted to its wards in each year, while some 130,000 persons are treated in the out-patients' department.

The hospital also maintains a Convalescent Home (70 beds), to which over 1,000 patients are admitted annually.

To carry on this great work involves an expenditure of about £75,000 a year, and until recently the revenues of the hospital were sufficient for its maintenance, and the general public was not asked to contribute in any way, but owing to the gradual expansion of expenditure consequent upon the advance of medical and surgical science, and to the reduction of the available income by some £9,000 a year, due to certain charges for interest and sinking fund in connection with the acquisition of additional site, there is now a deficit of about £12,000 per annum.

The capital debt upon which interest is payable is, approximately, £170,000, of which sum £50,000 was borrowed to meet current obligations mainly in connection with the recent structural additions.

While acknowledging the generous response accorded to the special appeal started in 1904 for re-building purposes, we feel very strongly that the claims of St. Bartholomew's Hospital have hitherto not been fully realised by the public, and it has consequently lacked support proportionate to its needs and to the great benefits it confers.

In again referring to the fact that the hospital has been maintained for generations, unaided by the public, we would mention incidentally that it has not participated in the benefactions of King Edward's Hospital Fund for London or the Hospital Sunday and Saturday Funds.

A special fund has been started to liquidate the capital debt and thereby reduce the annual deficit, and we very earnestly appeal for contributions for this purpose and for annual subscriptions towards the general maintenance of the hospital.

Cheques may be addressed to the Right Hon. the Lord Mayor, Mansion House, E.C., or to the Lord Sandhurst, Treasurer, St. Bartholomew's Hospital, London, E.C.

The following donations have already been received or promised, namely :—The Governor and Company of the Bank of England, £1,000; the Worshipful Company of Mercers, £500; the Worshipful Company of Merchant Taylors (in two instalments), £1,000; the Worshipful Company of Salters, £105; the Worshipful Company of Skinners (in five instalments), £1,000; the Worshipful Company of Ironmongers, £105; anonymous, £250.

We have the honour to be your obedient servants,

G. Wyatt Truscott (Lord Mayor), A. F. London, Sandhurst (Treasurer of the Hospital),

Aldenham, F. G. Banbury, Otto Beit, Blyth, Henry T. Butlin, W. S. Church, C. A. Cripps, Dyce Duckworth, R. B. Finlay, Ernest Flower, Claud Hamilton, Alex. Henderson, Arthur Hill, R. W. Inglis, E. Letchworth, John C. Lovell, J. H. Luscombe, Richard B. Martin, S. Hope Morley, R. Douglas Powell, Alfred de Rothschild, Marcus Samuel, Henry Schröder, Edgar Speyer, Thomas Sutherland, Edward P. Tennant, W. P. Treloar.

October, 1909.

